# Citizens’ Preparedness to Deal with Emergencies as an Important Component of Civil Protection

**DOI:** 10.3390/ijerph19020830

**Published:** 2022-01-12

**Authors:** Jozef Kubás, Katarína Bugánová, Mária Polorecká, Katarína Petrlová, Adéla Stolínová

**Affiliations:** 1 Department of Crisis Management, Faculty of Security Engineering, University of Žilina, 010 26 Žilina, Slovakia; Katarina.Buganova@uniza.sk; 2 Department of Fire Engineering, Faculty of Security Engineering, University of Žilina, 010 26 Žilina, Slovakia; Maria.Polorecka@uniza.sk; 3 Mathematical Institute in Opava, Silesian University in Opava, 74 601 Opava, Czech Republic; Katarina.Petrlova@math.slu.cz (K.P.); adelkaa.stolinova@seznam.cz (A.S.)

**Keywords:** security, safety, crisis management, risk management, emergencies, emergency preparedness, disasters

## Abstract

The main purpose of this paper is to point out a new approach in evaluating the preparedness of the population of a selected city for civil protection and its response to emergencies. Using new approaches, it evaluates a subjective questionnaire survey in combination with the objective state thanks to a mathematical approach and its subsequent verification on a specific example. The proposed approaches are then verified by experimental surveys in the selected city. The result is a highly adaptable tool that can be set up and adapted to different situations and different types of questionnaires to address the preparedness and safety of the population for emergencies. Thanks to this tool, it is possible to evaluate the subjective opinions of the population and thus gain insight into the assessment of the city’s preparedness for emergencies. Subsequently, we can set the prevention and preparedness of the population in the city on the basis of the obtained outputs, which potentially has a fundamental influence on the response after the occurrence of an emergency. Improving preparedness in the area of civil protection shall not only be reflected in the response and minimization of the consequences of the emergency, but also in the emotional security of the population.

## 1. Introduction

The preparedness of the population to deal with emergencies is now of growing importance. The increasing population density, growing dangers (anthropogenic events—accidents, increasing frequency of emergencies), and urban growth call for risk analysis strategies (including hazard, exposure, and vulnerability factors) in urban areas [1,2]. At the same time, it is necessary to ensure that citizens respond adequately to emerging emergencies. Pirlon (2020) states that local authorities need to make the necessary changes to meet the future challenges by reducing the vulnerability of people and the urban environment [3]. The role of cities is even more important given that more than a half of the world’s population lives in urban areas. According to the UN, by 2050, up to 68% of the world’s population will live in cities [4].

Some authors state that according to the Focus on Urban Risk of International Federation of Red Cross and Red Crescent Societies, cities and municipalities should pay more attention to the most vulnerable social groups when implementing their disaster risk reduction policies in areas of urban development and expansion (namely in poor and marginalized groups) in order to avoid structural and socio-economic barriers [5,6]. Moisidi (2018) states that the EU civil protection legislation has placed more emphasis on disaster prevention and preparedness since 2013, with a particular focus on risk assessment and risk management planning [7].

The risk is the probability that some results will have a negative impact on people, systems, or assets, which is usually displayed as a function of the combined effects of hazards, assets, or people at risk and the vulnerability of these exposed elements [8,9]. The concept of risk is closely related to the concept of danger, as the risk consists of danger and vulnerability, i.e., it depends on the intensity of the danger and the level of vulnerability. Dangers can have different impacts on individuals, groups in a society, or certain areas, e.g., urban regions. Vulnerability determines the level of impact intensity, and it can be further divided into exposure and manageability [10,11,12].

As disaster risks are strongly linked to social vulnerability, the impact assessment needs to be looked at from the societal perspective [13,14,15]. Research that examines the socio-psychological aspects of emergency management reveals much about non-technical/rational-analytical factors that affect individual and community preparedness, emergency decisions, warnings, and evacuation responses [16]. The role of the local population is topical for local disaster risk reduction because, despite socio-economic constraints, the local people can respond, recover, and deploy activities to face emergencies [17,18].

Much literature is devoted to examining risk perceptions and previous experience in influencing public evacuation behavior, and the link between the risk awareness and preparedness, informing the public about scientific uncertainty and the likelihood of risk, drafting a risk report, and so on [19]. It is necessary to know what the population perceives as a risk and what type of information they need. This information must be related to the specific risk of the particular area [20]. Measures to prepare, prevent, and adapt these provisions are based on the emergencies themselves; it is necessary for the population to be clearly informed about the risks to which they are exposed [5].

Ainuddin (2012) argues that risk awareness and preparedness can affect people’s vulnerability, as poor cognizance on their part can significantly worsen their vulnerability [21]. Preparedness can be understood as the knowledge and capabilities developed by entities (individuals and organizations) to effectively anticipate, respond to, and recover from the effects of disasters. Disaster awareness and household preparedness are key to reducing the negative effects of a disaster [22].

In the context of disaster risk management, disaster risk is influenced by broader national and global factors; however, it is formed at the local level [23]. There are no initiatives, policies, or strategies in Slovakia in the area of increasing the resilience of its society to disasters. Similarly, there is a lack of initiatives that would lead to any strategic development of the population’s preparedness in this respect. The training of young people (university students) and adults for disaster protection is carried out (only to a limited extent) by district authorities, legal entities, and natural persons—entrepreneurs [24,25].

International experience shows that campaigns have emphasized the importance of disaster kits. For example, the Australian government has guidelines for emergency kits, updates of alerts, and warnings; it also carries out disaster education through schools and ongoing research. The American government holds a ‘Get10′ campaign that publicizes a disaster kit. The Canadian government provides guidelines for household emergency kits, and it organizes the national Emergency Preparedness Week annually to promote emergency preparedness through local events and media coverage. In Nepal, organized training programs and guidelines are provided for the preparation of emergency kits and family emergency planning [26,27,28,29,30]. Despite the important role played by the National Civil Protection Organization, several case studies in Italy from north to south show that communication and cooperation between institutions and citizens is at a high level only immediately after the disaster [5,31,32,33]. Nevertheless, the focus on the places and the people affected tends to gradually fade and then completely disappear until the next disaster occurs [34].

To protect life, health, and property in the event of an emergency, it is necessary to analyze the potential threat, take measures to reduce the risks of the emergency, and to identify procedures and actions in dealing with the consequences of such emergencies [35]. In this whole process, it is necessary to focus on the opinion of the population and their preparedness to handle emergencies as well as use their opinions to improve it. Several scientific papers emphasize the need to examine these views. They also emphasize the need to further use the views and thus improve the level of the population preparedness by means of various tools [36,37,38]. Germany, for instance, is aware of the use of the potential of the population and their views, where the Federal Office for Civil Protection and Disaster Relief under Section 4 of the Civil Protection and Disaster Relief Act has significantly strengthened and developed the social science perspective in civil society protection in recent years. The socio-scientific dimension of crisis management will be further developed as a situational picture of the population’s behavior and will be more significantly implemented in risk prevention. One of the main topics is how to possibly use scientifically proven knowledge about the behavior of the population, its (information) needs, and self-defense capabilities in crises and disasters for decision-making processes in crisis management [39]. There are several approaches to obtaining such an opinion correctly. Some authors use the Person-Relative-to-Event theory model to assess the readiness of the population, with which they support their research claims [40]. Other authors, by contrast, point at the possibility of using The Protective Action Decision Model to examine threat perception [41]. The most common problems in applying these methods and approaches are the complexity of their use as well as their low informative value. Therefore, an absence of the use of specific opinions of respondents occurs quite often. As a result, it is important to develop an appropriate metric that can be used to assess the current state of preparedness of the population. When obtaining respondents’ opinions, it is very important to ask questions correctly. They must be comprehensible and have the necessary expressive value. The questions should focus on the identification of respondents, their subjective opinions, theoretical knowledge, and on the possibilities of improving their preparedness.

According to Act 42/1994 on Civil Protection, a natural person is entitled to an early warning of an imminent danger and immediate assistance in endangering his/her life, health, and property; he/she has the right to evacuate and hide, and to be informed about the method of protection. They also have the right to create the conditions to provide civil protection training, the aim of which is to enable them to acquire the necessary knowledge and skills for protection and to help others in need [25]. Our area of interest is the preparation of the population for self-protection, which is ensured through the Decree of the Ministry of the Interior No. 303/1996. It understands the provision of preparation for civil protection and mutual assistance as a purposeful and continuous process of preventive-educational and promotional activities; theoretical as well as practical training enables individuals to acquire the necessary knowledge, skills, and habits for self-protection and assistance to others in need [42]. The main forms of population preparation for self-protection and mutual assistance are as follows [42]:

- information and advisory service;
- programs broadcasted on the radio and television;
- publishing activities (professional publications, brochures, puzzles, etc.);
- preventive-educational and promotional activities (exercises, competitions, exhibitions, and excursions);
- theoretical and practical training;
- publication of information in an electronic form.

In order to assess whether the preparation of a population is effective and at a sufficient level, it is necessary to determine the state of the knowledge and awareness of the population. Therefore, the aim of this article is to create a way to assess the preparedness of the population for emergencies and to replicate it in the selected municipality.

## 2. Materials and Methods

To increase the level of preparedness of the population for emergencies, it is necessary to be able to assess the current situation. For these needs, a simple tool has been developed to assess the total rate of the respondents’ preparedness for emergency. So, it is the basis for a purely political decision on a possible financial investment in the field of civil protection. This basis should be as simple and comprehensible as possible. This leads to the need for data aggregation, optimally up to a single number. Accordingly, the methodology of evaluation of the questionnaire is conceived—an illustrative method of using data aggregation was used.

The proposed instrument has several limiting conditions and expected errors (uncertainties). Sampling-related errors include data collection that has been performed under conditions that cannot be repeated, the non-existent method of selecting the respondents who answered the questionnaire and therefore there is no possibility to repeat the structure of the respondents, and the unrepresentativeness of the structure. The reason for the chosen sampling was time and economic savings. Non-sampling errors include the unreachability of respondents, their reluctance to communicate, data processing errors, data analysis errors, inaccurate answers from respondents, and difficulty quantifying.

These errors are present in every survey, regardless of sample size. The survey was used mainly to verify the metrics. The results are available to the city and serve to quickly verify the current state. For more accurate results, it is planned to allocate more funding and resources to replicate this research with an emphasis on representativeness. Such research should be repeated at regular intervals to compare how new measures and communication by the city are perceived by residents and how their readiness changes.

A questionnaire was developed to take advantage of the population’s views on security and their preparedness for emergencies. The questionnaire’s very form enables it to quickly and efficiently obtain answers from a number of respondents. The questionnaire consists of several types of questions. It is essential to identify the respondent, which will allow for comparing the answers in different groups and to propose measures with regard to the results of specific groups. Other types of questions focused on objective and subjective indicators. These issues were based on a generally binding legal regulation in Slovakia, which addresses the issue of civil protection of the population. Among others, they contain and describe the extraordinary events that may occur in the given area. In the event that the research takes place in another country, it is of course necessary to adapt the questions to its generally binding regulations concerning the given subject matter. The questionnaire is supplemented by a question that seeks an opinion on informing about the issues by the municipality and a question that focuses on the form of informing. It is the findings concerning the form of informing that will enable the municipality to better concentrate on specific groups and choose appropriate forms. The questions were closed, and some could be marked on the Likert Scale. In some, the possibility of “I do not know” (meaning the respondent cannot judge) is added so that the respondent is not forced to mark an answer about which he/she is not convinced.

The city of Žilina was selected to verify the questionnaire, where the questionnaire was assessed and shared on the official city website in cooperation with the city representatives. The questionnaire aimed at assessing the preparedness of the inhabitants of the city of Žilina for extraordinary events is shown in Table 1. The city of Žilina functions as the center of northwestern Slovakia and is the fourth largest city in the Slovak Republic. It is the seat of the bodies of the Žilina self-governing region, one of the eight regions of the Slovak Republic. It covers an area of 80.03 km^2^ and has a population of 82,494 inhabitants. It is located in the valley of the river Váh, in the Žilina Basin, at the confluence of Váh with the rivers Kysuca and Rajčianka. The basin is formed by Tertiary sediments—conglomerates with inserts of soft sandstones of the Carpathian Paleogene. Relatively wide river floodplains stretch along Váh and Rajčianka, accompanied on the sides by Pleis-Tocene gravel terraces. The Žilina Basin is located among the Malá Fatra, Strážovské vrchy, Súľovské vrchy, Javorníky, and Kysucká vrchovina mountains. The average air temperature in July reaches +18 °C, in January −4 °C. The annual average precipitation is 650 to 700 mm, mostly in June and the first half of July. The snow cover is 60 to 80 days a year [43]. In our opinion, what will make it possible to verify the new metric to determine the preparedness of the population for emergencies and to suggest ways to improve it is the research on a specific city.

The questionnaire survey during the primary data collection included those citizens who duly filled in and sent the completed questionnaire on the official website of the city of Žilina in the period from 1 July 2021 to 30 June 2021. The questionnaire was also shared through social networks and targeted the inhabitants of Žilina. The reason for collecting the necessary data was to verify the metric on the results of the questionnaire survey in a particular city. The intention was also to verify the suitability of the questions for research needs. The completed questionnaire survey meant no costs for the city and was not time-consuming. The disadvantage was that the obtained sample did not meet the conditions of representativeness. If the research is repeated, the city will need to set aside time and resources, among other things, to ensure the representativeness of the survey, so that the results are as relevant as possible. On the second level, only those questionnaires were processed and selected from the collected questionnaires, where the respondents answered the question number 1 as citizens of the city of Žilina. These circumstances define the first and second levels of inclusive and exclusive criteria. Therefore, the key to assessing the total preparedness of the inhabitants of the city of Žilina for emergency was the creation of a tool that will enable this preparedness to be “measured” on the basis of the data obtained from a questionnaire survey. That is, the creation of a metric that will make it possible to express the preparedness of the population in a simple and maximally comprehensible way, preferably with a single number. At the same time, however, it is necessary to obtain information on the structure of preparedness. Both sorts of data are needed in order for the management of the city of Žilina not only to know how prepared its inhabitants are for emergency, but also to obtain information on what to focus on so that their preparedness can be increased if necessary. The composition of the questions was based on generally binding regulations [25,44]. In the case of conducting surveys in other countries, it is appropriate to modify the issues according to the particular legal regulations. The metric is designed to be repeated in other municipalities and countries.

It is first necessary to introduce the appropriate quantities and define the appropriate metrics. To this end, the questions in the questionnaire were divided into several groups according to their nature. As a result, the key issues for determining total preparedness were divided according to their nature into two groups: objective and subjective. The distribution was chosen because of the complete assessment of total preparedness, which typically consists of both the objective knowledge of the population (e.g., relevant laws, decrees, and standards) and the fully subjective ability of each individual to respond appropriately to an emergency.

The objective questions (questions 9, 11–16) indicate only the innocence or ignorance of the corresponding theoretical knowledge in the field of emergency preparedness. Each correct answer was awarded one point in the evaluation and incorrect answers were awarded zero points. The maximum possible (non-standard) value for the objective criteria $O_{\max}$ is 10 points (question no. 11 has four sub-questions), while $O_{i}$ is the total achieved point value for the objective criteria for the *i*-th respondent.

The subjective questions (questions 5–8, 10, 17–18) indicate for the given respondent to what extent he/she is able to adequately respond to the given type of emergency and whether he/she is interested in the issue of civil protection. They thus express their subjective evaluation of their own preparedness. Maximum (non-standard) point value for the subjective criteria of the *i*-th respondent $S_{\max}$ is 17 points. $S_{i}$ is the total achieved point value for the subjective criteria of the *i*-th respondent.

As the questions are of a different nature, it was necessary to set the evaluation of the answers correctly. The evaluation of individual answers is given in Table 2.

Before using the mathematical apparatus itself, it is necessary to define the quantities. All quantities gradually used in the mathematical apparatus are shown in Table 3.

The introduction of these quantities was a necessary step in the correct definition of both metrics P and R. Metrics P and R are functions of $S_{i}$ and $O_{i}$: $P=P\left(S_{i},O_{i}\right)$, $R=R\left(S_{i},O_{i}\right)$. Each of the quantities $S_{i}$ and $O_{i}$ has a different point range, so it is advisable to standardize them first. Therefore, “standardized” quantities ${\bar{S}}_{i}$ and ${\bar{O}}_{i}$ are introduced:

$${\bar{O}}_{i}=100\frac{O_{i}}{O_{max}},$$

$${\bar{S}}_{i}=100\frac{S_{i}}{S_{max}},$$

where ${\bar{S}}_{i},\;{\bar{O}}_{i}\in I_{100}=\langle 0\;\mathrm{pts}.,\;100\;\mathrm{pts}.\rangle Ɐi\in \left\{1,\ldots ,n\right\}=I_{n}$.

The set of quantities ${\bar{S}}_{i}$ and ${\bar{O}}_{i}$ forms a coordinate system on the space $V=I_{100}\times \cdots \times I_{100}=I_{100}^{2n}$. It is now possible to introduce new coordinates $P_{i}$ and $R_{i}$ in space V:

$$P_{i}=\frac{1}{2}\left({\bar{S}}_{i}+{\bar{O}}_{i}\right),$$

$$R_{i}=\frac{1}{2}\left({\bar{S}}_{i}-{\bar{O}}_{i}\right)+\frac{\max\left\{I_{100}\right\}}{2}.$$

The last constant term on the right side of the second of the above relations shifts the domain from the interval ⟨−50 pts., 50 pts.⟩ to the interval ⟨0 pts., 100 pts.⟩. If this term were not there, it would not be coordinates on space V. The $P_{i}$ coordinate indicates the overall degree (subjective and objective) of the *i*-th respondent’s emergency preparedness. The $R_{i}$ coordinate indicates the total difference rate (subjective and objective) of the *i*-th respondents emergency preparedness.

The metric P is defined as the mean. The sets of values ${\left\{P_{i}\right\}}_{i\in I_{n}}$ and ${\left\{R_{i}\right\}}_{i\in I_{n}}$ can be understood as the realization of two random variables. Therefore, it makes sense to determine the numerical characteristic of these random variables (e.g., mean or variance) or to verify that they have the character of some known type of data distribution of all $P_{i}$:

$$P=E\left({\left\{P_{i}\right\}}_{i\in I_{n}}\right)=\frac{1}{2n}\sum_{i=1}^{n}\left({\bar{S}}_{i}+{\bar{O}}_{i}\right).$$

The metric R is now defined as the mean of all $R_{i}$:

$$R=E\left({\left\{R_{i}\right\}}_{i\in I_{n}}\right)=\frac{1}{2n}\sum_{i=1}^{n}\left({\bar{S}}_{i}-{\bar{O}}_{i}\right)+\frac{\max\left\{I_{100}\right\}}{2}.$$

Metric P represents the total rate (subjective and objective) of the respondents’ preparedness for emergency. The interpretation of their significant values is as follows:

- P=0 pts.—the respondents’ complete unpreparedness for emergency,
- P=50 pts.—the respondents’ half-preparedness for emergency,
- P=100 pts.—the respondents’ complete preparedness for emergency.

Metric R represents the total difference rate of the respondents’ preparedness for emergency. The interpretation of their significant values is as follows:

- R=0 pts.—purely subjective preparedness for emergency (no objective preparedness);
- R=50 pts.—the same subjective and objective preparedness for emergency;
- R=100 pts.—purely objective preparedness for emergency (no subjective preparedness).

Furthermore, for 0 pts. ≤R<50 pts., the objective respondents’ preparedness for emergency prevails over their subjective one. By contrast, for 50 pts.<R≤100 pts., the subjective respondents’preparedness prevails over their objective one.

The main motivation for the creation of *P* and *R* metrics was the inclusion of objective and subjective criteria in the simplest possible form and, moreover, so that, if necessary, the distribution of “importance” between the objective and subjective criteria could be easily adjusted. Another motivation for the creation of the *P* and *R* metrics in this form was the comprehensibility of the total evaluation and the related easy interpretability of the obtained results. For this reason, the whole concept is created in such a way that both metrics take values from the interval ⟨0 pts., 100 pts.⟩, and the same importance was set for subjective and objective criteria. This is also evident from the fact that the relations for $P_{i}$ and $R_{i}$ have the same coefficient ½ for the quantities ${\bar{O}}_{i}$ and ${\bar{S}}_{i}$. Consequently, both quantities have the same “weight”. Weight adjustment—the adjustment of the importance of subjective and objective criteria—is then performed by adjusting the coefficients in the relationships for pro $P_{i}$ and $R_{i}$. The coefficients should be chosen so that their sum is equal to one, as in the case described above.

The evaluation of *P* and *R* metrics, while applying the selection criteria from questions 2–4 (see Table 1) and their mutual comparison, will provide additional information about the total preparedness and differential preparedness of respondents for emergency for groups of respondents meeting individual criteria (gender, age, education).

One of the possible problems in assessing the results of the questionnaires is the problem of the representativeness of the set of respondents. This problem is relatively complex, so in order to assess the representativeness of the sample of the population of the city of Žilina, space was devoted to questions of a classification nature (questions no. 1–4). Based on the answers to question no. 1, those respondents who stated a residence other than the city of Žilina were excluded. By evaluating the answers to questions 2–4, it is possible to determine whether at least the necessary, but not sufficient, conditions for the representativeness of the sample of the examined population are met.

To obtain additional information, the questionnaire lists questions 19 and 20, which are semi-open and open. Their purpose is to obtain additional information on the views of respondents on the preferred ways of being informed about civil protection and population protection.

## 3. Results

The first questions made it possible to identify the respondents. Question 1 of the questionnaire serves as an exclusion criterion. Only the part of the respondents who stated in the questionnaire that they were from Žilina was included in the calculations. A total of 340 respondents took part in the questionnaire survey, while there were 316 respondents from Žilina, which is about 93% of all respondents. The basic results obtained from these questions of the questionnaire are shown in Table 4.

The key information about the set of obtained data needed to assess the total preparedness of the inhabitants of the city of Žilina for extraordinary events consists of sets of values ${\left\{P_{i}\right\}}_{i\in I_{n}}$ and ${\left\{R_{i}\right\}}_{i\in I_{n}}$. Where $P_{i}$ is the total rate (subjective and objective) of the preparedness of the *i*-th respondent for emergency and $R_{i}$ is the total difference rate (subjective and objective) of the preparedness of the *i*-th respondent for emergency.

The most fundamental quantity for the total assessment of the preparedness of the population of the city of Žilina for emergency is the metric *P*, hence the total rate (objective and subjective) of the respondents’ preparedness for emergency. An additional quantity is the metric *R*, hence the total difference rate (subjective and objective) of the respondents’ preparedness for emergency. These and other numerical characteristics for ${\left\{P_{i}\right\}}_{i\in I_{n}}$ and ${\left\{R_{i}\right\}}_{i\in I_{n}}$ are listed in Table 5.

Table 4 and the method of introducing metrics P and R show that:

P=39.89 points,

R=46.22 points. 

Metric P, hence the total rate (subjective and objective) of the respondents’ preparedness for emergency, was defined as the mean value of all $P_{i}$. From Table 4, it becomes obvious that the value of metric P, is 39.89 points. According to the proposed evaluation in Chapter 2, the result falls within the interval between the complete respondents’ unpreparedness for emergency and the respondents’ half-preparedness for emergency. The resulting value is closer to respondents’ half-preparedness for emergency. The result can generally be interpreted as a relatively low rate of total emergency preparedness. According to Table 4, the *R* metric, hence total difference rate (subjective and objective) of the respondents’ emergency preparedness, acquires the value of 46.22 points, which, according to the evaluation from Chapter 2, is in the interval between completely objective emergency preparedness and the same subjective and objective emergency preparedness. The result can be interpreted in such a way that the objective preparedness of respondents slightly prevails over the subjective one. Another authoritative data is the variance of $P_{i}$ values, which is 203.77 pts^2^. It can be interpreted as rather higher, i.e., there is a relatively large difference among the knowledge of individual respondents. From the value given in the upper quartile column, it shows that 75% of respondents achieved a score below 50 points. The proximity of the mean and median of all $P_{i}$ indicates that the distribution of data is almost symmetric. The maximum achieved value of $P_{i}$ is 77.06 points, which means that the best of the group of respondents does not reach even 80% preparedness for emergency.

Other information that can be obtained from the questionnaire survey is the information about metrics P and R, that is the total rate (objective and subjective) of the respondents’ emergency preparedness for groups respondents by gender, age, and education (see Table 1, questions 2–4). The values are given in Table 6.

Due to age, the metric *P* is relatively balanced in all categories. The only group showing an above-average value of the total rate of the emergency preparedness is the aged 60 and over. At the same time, the objective preparedness clearly outweighs the subjective preparedness in this group (metric *R*). Subsequently, the age category over 60 years feels the least subjectively prepared for emergency, but in the total result it performs above average in their objective knowledge. In terms of gender, the total rate of emergency preparedness is higher for the male according to the results, which can be explained by a higher level of subjective feeling of preparedness, as shown by metric *R*. Depending on education, the category of secondary school without the graduation exam deviates significantly, where the metric P is 30.77 points, which is the lowest value of all groups. The category of secondary schools can be understood as a remote value, while the trend form metrics *P* then increases with education for the remaining values. The subjective emergency preparedness of the remaining groups decreases with an increasing education at the expense of the objective one. These data can be valuable in targeting additional education of the population by the representatives of the city of Žilina in order to improve the resulting total score of emergency preparedness.

Questions no. 2–4 are mainly used for a possible assessment of the representativeness of the set of respondents as a sample of the population of the city of Žilina (see Table 1). In order for the result of the metric P to have a completely undistorted and meaningful value, it is necessary to ensure the representativeness of the obtained data set. From the data on gender distribution, it can already be concluded that the necessary condition of representativeness is not met. Assuming that the questionnaire was filled in more by those interested in the given issue, from whom a higher level of knowledge about the issue can be expected, it can be concluded that the real total rate of the emergency preparedness will actually be lower than found. Therefore, it can be expected that the value of the total determined rate of the emergency preparedness of the inhabitants of the city of Žilina will be the upper limit of the real value of this preparedness. Although the representativeness of the population sample was not accurately maintained, the data obtained can still be used to evaluate and test the proposed *P* and *R* metrics with the above limitation. The result is the motivation to adjust the methodology of data acquisition so that the necessary conditions for the representativeness of the obtained data set are met.

## 4. Discussion

The article focuses on a new approach in evaluating the preparedness of the population for civil protection and its response to emergencies. The proposed approach is then verified by experimental surveys in the Žilina city. Thanks to this tool, it is possible to evaluate the subjective opinions of the population and thus gain input to the assessment of the city’s preparedness for emergencies. Based on the obtained outputs, it is possible to set the prevention and preparedness of the population in the city by the representatives of the Žilina city, which has a fundamental influence on the response after the occurrence of an emergency.

The research results show some important information for the city of Žilina. The results of the survey describe the relatively low level of emergency preparedness (P = 39.89 points out of 100) of the inhabitants of the city of Žilina. The city of Žilina should therefore pay attention to increasing the level of population prevention education of its inhabitants, for example, by targeting the groups that showed the weakest results in the questionnaire. Although the sample examined did not meet the criterion of representativeness, the information obtained can still be very valuable for the city, as it will help the city to obtain a basic overview of the views of its residents on the issue in a short time. The crisis management staff of the city of Žilina has the results of the investigation at their disposal and will use them to improve the preparedness of the population to deal with emergencies. We expect the research to be repeated in the future. Emphasis will be placed on the representativeness of the sample in order to obtain most relevant information needed to improve the preparedness of all citizens for various emergencies.

Just over 2.42% of respondents are convinced that the city of Žilina provides sufficient information on civil protection and population protection (question 19). In the questionnaire survey, as many as 32.02% of the respondents stated that the city of Žilina tends to inform its inhabitants insufficiently in the field of civil protection and population protection. The highest percentage of respondents shared that they did not know whether informing on the part of the city was sufficient. The results of the survey on informing the city of Žilina about civil protection and population protection are shown in Table 7.

The results of question 20 indicate that the respondents stated the creation of a transparent website with all the necessary information (24,69%) as the most preferred form of information, followed by Facebook and Instagram (20.22%) and an information leaflet (15,30%). The city of Žilina should focus mainly on creating a clear and concise website that would inform the inhabitants of the city about the basics of population protection and, most importantly, how they should behave in the event of specific emergencies threatening the region. The respondents lack comprehensive information on the immediate response to individual extraordinary events, which would be available on the website of the city of Žilina. Younger respondents would also appreciate the information on social networks. Still, it is questionable how to set the content of the reports on the protection of the population so that they are not completely lost in the flood of much more interesting daily news. The fact that respondents often follow social networks does not mean that they look for practical information on them. When creating an information leaflet, it is essential to solve the distribution to individual households in particular and to choose such a visual form that the inhabitants of the city would be attracted, and the leaflet will not end up unnoticed directly in the sorted waste. In comparison, book publications (2.12%), courses or trainings (6.15%), and lectures (5.70%) are the least sought-after forms of information int the field of civil protection and population protection. The respondents show minimal interest in these forms of education in the field of population protection. It is probably not very important for the city of Žilina to invest time and money in the least required forms of education and, conversely, it should focus on the forms that the respondents preferred according to the questionnaire survey instead. The specific results are shown in Table 8.

When asked about the preferred form of education, the respondents were also given an opportunity to verbally complete the missing form. Four respondents took the opportunity and recommended a mobile phone application, billboards, or a clear guide for each household. One respondent pointed to the need for an easy-to-understand language for scholars or people with disabilities.

Due to the age of the respondents, the age group of 60 and over reached the above-average value of the total rate of the emergency preparedness. The reason for the result may be, among other things, the fact that the given age group regularly completed the so-called military education as a compulsory part of school attendance in the past. However, for example, official sources of the Fire and Rescue Service of the Czech Republic (HZS ČR) show that seniors tend to be the most vulnerable group in emergencies, due to their reduced mobility or slower evaluation skills [45]. As a result, since 2010, the HZS ČR has decided to pay more attention to this group of the population than before. A sufficient amount of free time, the desire to learn something new, the willingness not to be indifferent to their surroundings, and enough life experience predispose the group of seniors to a long-term and quality education in the field of civil protection and population protection.

In connection with education, the categories of secondary education without the graduation exam deviate from the results of the questionnaire survey. The respondents from this group achieved the lowest rate of preparedness for emergencies. If the city of Žilina is interested in increasing the level of emergency preparedness of the population, it should start with this category and can use the results of the last question of the questionnaire survey. In the question, the respondents directly identified those forms of information about civil protection and population protection that are close to them, i.e., those which they would prefer themselves. The obtained results of the preferred form of information on civil protection and population protection for the group of respondents from the category of secondary education without the graduation exam are shown in Table 9.

A comparison of Table 7 and Table 8 clearly shows that the respondents belonging to the category of secondary education without the graduation exam prefer the same forms of information as all other respondents, regardless of the classification of respondents into groups according to education. As a matter of fact, if the city of Žilina decides to raise the awareness of its concerns, it should obviously start by creating a transparent website with all the necessary information in the field of civil protection and population protection. This information should also serve as a potential basis for other municipalities that address similar issues.

The municipality plays an important role in reducing the risk of disasters and emergencies, as it serves as the primary point of contact for its population in terms of subsidiarity and the ability to build resilience. The content and form of preparedness of the population for emergency is chosen by the municipality itself [24,42]. A lack of human and technical resources leads to poor emergency awareness and ineffective implementation of prevention and mitigation strategies at the local level [46].

Higher income and education levels appear to be important indicators of development that can reduce vulnerability and enable citizens to engage in self-protection [47,48]. Education increases people’s knowledge about disaster risks and influences their risk perception [49,50]. In addition, public awareness of disaster risk reduction is a key factor influencing their behavioral decisions [51]. However, the communication gap between professionals and lay public can be a problem. There is a need for local authorities and experts to establish the conditions for spreading a culture of awareness of the risks of a given area and to increase the level of security of a particular area through concrete and participatory actions (bottom-up approach). However, communication about natural disasters or emergencies does not only mean informing citizens, but also assessing whether they understood the content of the communication. In fact, everyone who communicates should necessarily be “aware” and informed about the needs and requirements of the community, as well as their level of understanding [20]. Therefore, attention needs to be paid to how people interpret the risks that shape their own experiences, feelings, values, cultural beliefs, and interpersonal and social dynamics [16]. Proper preparation of the population for various unexpected events will increase their safety and can positively influence the response to emergencies and disasters. Consequently, it is necessary for municipalities to know the current state of preparedness of the population and take measures based on them. Such activities can also improve the security environment in which residents find themselves. In the event of a crisis, the prepared population reflects the preparedness of the emergency for various emergencies and disasters [52,53,54].

What is more, disaster awareness research may indicate a weak or poor perception of the links among people in a given area or may help to clarify the lack of knowledge among the people in a given area [55,56]. In addition to extensive research into disaster risk perceptions and awareness, research is often tied to local conditions. This is due to the specific conditions that endanger the population as well as to the generally binding regulations in the area under assessment [57,58]. Donahue et al. pointed out that local government officials often do not know the views of the population. Another problem is the low informative value of the opinions obtained or the incorrect use of opinions [59]. Appropriately obtained and evaluated opinions of the inhabitants will enable the local government managers and municipalities to adapt the information and educational process so as to improve the readiness of the inhabitants themselves. It is also important to be able to calculate the preparedness of the population, which will allow municipalities to compare how the implemented changes affect the preparedness.

## 5. Conclusions

In the article, we introduced a new tool that could be used to assess the level of preparedness and awareness of the population in a municipality for emergencies. This tool is a new metric that can be applied to the needs of other municipalities too. The result of the metric is a value that expresses the level of preparedness of the inhabitants of a municipality. The views of the population are included in this metric, which can be obtained by the method of a questionnaire. The questionnaire we created serves the environment of Slovak municipalities, and, in case of repetition in other countries, it should be adjusted according to local conditions. Based on the results, the municipality can determine the right way to further increase information and provide training of the population in this respect. We verified the proposed means in the city of Žilina, where we pointed out the possibility of verifying the metric in practice. The overall survey and focus of the article are in accordance with the requirements of the legislation of the Slovak Republic [25]. The approach chosen is therefore closely linked to the regional conditions, legislation, and customs in the country and therefore needs to be adapted to the conditions in the country that would possibly use this approach.

Based on the proposed calculations, we can draw clear conclusions from the above stated facts that the city of Žilina and its inhabitants are not sufficiently prepared to respond in the event of various types of emergencies. It is very important to realize that the assessment of this preparedness is based not only on objective facts, but especially on the subjective assessment of the population concerned. Thanks to the presented tool that can be used to evaluate the subjective view of the population in combination with objective factors, we can realistically determine the state of preparedness and also propose adequate measures on that basis. By further developing and adapting the proposed tool, it is possible to respond flexibly and adequately to the state of knowledge and preparedness of the population and thus significantly streamline prevention in the field of civil protection. The point is that a well-informed and educated population in response to an emergency can reduce the burden on rescue services and crisis managers. Moreover, it will also help reduce the consequences of an emergency and thus contribute to the overall security. These facts will also affect the emotional security and quality of living of the population.

## Figures and Tables

**Table 1:** Questions from the questionnaire.

No.	Question	Answer
1	Place of residence	Žilina/Other
2	Age	
3	Sex	Female/Male
4	Highest level of education	Primary/Secondary without the graduation exam/Secondary with the graduation exam/Bachelor’s degree/Master’s degree/Doctoral degree
5	Do you think that you can react appropriately in the event of the following natural disasters?	
	Floods and inundations	Yes/Rather yes/Rather no/No/I do not know
	Hailstorm	
	Swelling	
	Landslides	
	Snow calamities and avalanches	
	Extensive icing	
	Earthquakes	
6	Do you think that you can react appropriately in the event of the following accidents?	
	Fires and explosions	Yes/Rather yes/Rather no/No/I do not know
	Leaks of hazardous substances, preparations, and wastes, petroleum products with subsequent contamination of land, air, watercourses, drinking water sources, and groundwater	
	Damage to distribution lines, their equipment, and transmission lines	
7	Do you think that you can react appropriately in the event of the following disasters?	
	Major air, rail, ship, and road accidents associated with fires or leaks of hazardous substances	Yes/Rather yes/Rather no/No/I do not know
	Nuclear accident	
	Damage to water structures	
8	Do you think that you can react adequately in the event of a terrorist attack?	Yes/Rather yes/Rather no/No/I do not know
9	The current threats to public health of the second degree in COVID-19 coronavirus SARS-CoV-2 is due to:	The occurrence of a communicable disease, suspicion of a communicable disease or suspicion of death of a communicable disease above the expected level/Release of chemicals endangering life, health, environment, and property/Leakage of microorganisms or toxins from enclosed spaces
10	Do you think that you can react appropriately in the event of a threat to public health of the second degree?	Yes/Rather yes/Rather no/No/I do not know
11	The population is warned by warning signals. Do you know what signal the sirens are announcing?	
	General threat	Two-minute fluctuating tone/Six-minute steady tone/Two-minute steady tone/I do not know
	Water threat	
	The end of the threat or the end of the effects of the emergency	
	Testing the operability of population warning systems after informing the population about the time of the test through the mass media	
12	The evacuation is announced through the mass media and is revoked if the reason for which it was announced has passed. Evacuation is divided into:	A short-term evacuation with a possible return of the evacuees within 24 h and a long-term evacuation with a possible return of the evacuators after 24 h/A short-term evacuation with a possible return of the evacuators within 48 h and a short-term evacuation with a possible return of the evacuators after 48 h/A short-term evacuation with a possible return of the evacuators within 72 h and a long-term evacuation with a possible return of the evacuators after 72 h/I do not know
13	The weight of the evacuation baggage may be at most:	15 kg + 5 kg of hand luggage for an adult; 5 kg + 5 kg of hand luggage for a child/25 kg + 5 kg of hand luggage for an adult; 15 kg + 3 kg of hand luggage for a child/25 kg + 5 kg hand luggage for an adult; 15 kg + 5 kg hand luggage for a child/I do not know
14	Do you know what the population protection plan is?	A document containing tasks, measures, and procedures to ensure the protection of the population in the event of an emergency/A document containing a plan for the population how to protect themselves/A document containing the description of all emergencies in the municipality with a proposal for a better solution/I do not know
15	The resident has the right:	To an early warning of an imminent danger, to evacuation and concealment, to information about the method of protection, and to an immediate assistance in case of danger to life, health, and property/Only to an immediate assistance in case of danger to life, health and property/Only for early warning of imminent danger and for evacuation or hiding/Do not know
16	Do you know where there is a space in your area for people to hide (the so-called civil protection cover)?	Yes/No
17	To what extent are you interested in civil protection and population protection on your own initiative?	I am intensely interested, I look for professional publications and news in this area/I am interested, I look for interesting things on the Internet and in the media/I am passive, I only accept information from the media/I am not interested at all
18	Have you seen or searched for information on civil protection and population protection at (you can also mark more than one answer):	The website of the city of Žilina/The website of the higher territorial unit of Žilina/The website of the Ministry of the Interior of the Slovak Republic/I have not seen any of these/I have not searched/Other—please specify
19	Do you think that the city of Žilina sufficiently informs its inhabitants about civil protection and population protection?	Yes/Rather yes/Rather no/No/I do not know/ Other—please specify
20	What form of information about civil protection and population protection would you prefer (it is possible to indicate more than one answer):	Creation of a clear website with all the necessary information/Courses or trainings/Lectures/Information leaflets/Available book publications/On the local TV or in the local newspapers/On Facebook, Instagram/None/Other—please specify

**Table 2:** The evaluation of individual answers.

Question No.	Answer	Value
9, 11, 12, 13, 14, 15 16	Correct answer	1
	Not right answer	0
5, 6, 7, 8, 10	Yes	1
	Rather yes	0.5
	Rather no	0.25
	No; Do not know	0
17	I am intensely interested	1
	I am interested	0.5
	Passive	0.25
	I am not interested	0
18	Any answer	1
	No answer	0

**Table 3:** Qualification of the quantities used.

Quantity	Symbolized by	Note
Number of respondents	N	n∈N
*i*-th respondent	i	$i\in \left\{1,\ldots ,n\right\}$
Maximum value of subjective criteria for a given respondent	$S_{\max}$	$S_{\max}=17\;\mathrm{pts}.$
Maximum value of objective criteria for a given respondent	$O_{\max}$	$O_{\max}=10\;\mathrm{pts}.$
The total achieved value of the subjective criteria of the *i*-th respondent	$S_{i}$	$S_{i}\in \langle 0\;\mathrm{pts}.,\;S_{\max}\rangle$
The total achieved value of the objective criteria of the *i*-th respondent	$O_{i}$	$O_{i}\in \langle 0\;\mathrm{pts}.,\;O_{\max}\rangle$
Total rate of the preparedness of the *i*-th respondent for emergency	$P_{i}$	$P_{i}\in \langle 0\;\mathrm{pts}.,100\;\mathrm{pts}.\rangle$
Metric—the total rate of the respondents’ preparedness for emergency	P	P∈⟨0 pts.,100 pts.⟩
The total difference rate of the preparedness of the *i*-th respondent for emergency	$R_{i}$	$R_{i}\in \langle 0\;\mathrm{pts}.,100\;\mathrm{pts}.\rangle$
Metric—the total difference rate of the respondents’ preparedness for emergency	R	R∈⟨0 pts.,100 pts.⟩

**Table 4:** Results of sorting questions.

Respondents	From Žilina	Outside Žilina					Total
Number [%]	92.94	7.06					100
Age Number [%]	<20	20–29	30–39	40–49	50–59	60<	Total
	4.43	43.04	26.58	16.77	5.38	3.80	100
Sex	Female	Male					Total
Number [%]	58.86	41.14					100
Education	Primary	Secondary without the graduation exam	Secondary with the graduation exam	Bachelor’s degree	Master’s degree	Doctoral degree	Total
Number [%]	3.78	4.11	50.32	12.97	23.73	5.06	100

**Table 5:** Basic numerical characteristics ${\left\{P_{i}\right\}}_{i\in I_{n}}$ and ${\left\{R_{i}\right\}}_{i\in I_{n}}$.

Quantity	Minimum [pts.]	Lower Quartile [pts.]	Median [pts.]	Upper Quartile [pts.]	Maximum [pts.]	Mean Value [pts.]	Variance [pts^2^]
${\left\{P_{i}\right\}}_{i\in I_{n}}$	7.21	29.08	38.38	49.12	77.06	39.89	203.77
${\left\{R_{i}\right\}}_{i\in I_{n}}$	17.50	39.12	46.18	52.68	79.85	46.22	124.63

Note: $P_{i},R_{i}\in \langle 0\;pts.,\;100\;pts.\rangle Ɐi\in \left\{1,\ldots ,n\right\}$.

**Table 6:** Metrics P and R for individual sorting characteristics.

Age [Years]	<20	20–29	30–39	40–49	50–59	60<
*P* [pts.]	39.23	40.46	39.98	38.53	37.23	43.37
*R* [pts.]	53.52	47.30	44.51	44.38	51.35	38.37
Sex	Female	Male				
*P* [pts.]	38.48	41.92				
*R* [pts.]	44.71	48.38				
Education	Primary	Secondary without the graduation exam	Secondary with the graduation exam	Bachelor’s degree	Master’s degree	Doctoral degree
*P* [pts.]	40.33	30.77	39.04	41.51	40.97	46.22
*R* [pts.]	53.66	46.15	46.65	45.65	45.24	42.47

Note: P, R∈⟨0 pts., 100 pts.⟩.

**Table 7:** Respondents’ sense of awareness of the city of Žilina in the field of civil protection and population protection.

Question	Answer				
	Yes	Rather Yes	Rather No	No	I Do Not Know
Do you think that the city of Žilina sufficiently informs its inhabitants about civil protection and population protection?	2.42%	9.06%	32.02%	21.75%	34.75%

**Table 8:** Preferred form of information about civil protection and population protection.

Preferred Form of Information	Percentage
1. A transparent website	24.69%
2. Courses or training	6.15%
3. Lectures	5.70%
4. Information leaflets	15.30%
5. Available book publications	2.12%
6. On the local TV or radio	13.97%
7. In the local newspaper	11.17%
8. Facebook, Instagram	20.22%
9. None	0.68%

**Table 9:** Preferred form of information about civil protection and population protection in the category of secondary education without the graduation exam.

Preferred Form of Information	Percentage
1. A transparent website	26.92%
2. Courses or training	7.69%
3. Lectures	3.85%
4. Information leaflets	15.38%
5. Available book publications	3.85%
6. On the local TV or radio	15.38%
7. In the local newspaper	11.54%
8. Facebook, Instagram	15.38%
9. None	0%

## Data Availability

The data presented in this study are available on request from the corresponding author.
